# A histological and diceCT-derived 3D reconstruction of the avian visual thalamofugal pathway

**DOI:** 10.1038/s41598-024-58788-z

**Published:** 2024-04-11

**Authors:** Parker J. Straight, Paul M. Gignac, Wayne J. Kuenzel

**Affiliations:** 1 Poultry Science Department, University of Arkansas, Fayetteville, AR USA; 2 Cellular and Molecular Medicine Department, University of Arizona Health Sciences, Tucson, AZ USA; 3 MicroCT Imaging Consortium for Research and Outreach, University of Arkansas, Fayetteville, AR USA

**Keywords:** Neural circuits, Sensory processing, Visual system

## Abstract

Amniotes feature two principal visual processing systems: the tectofugal and thalamofugal pathways. In most mammals, the thalamofugal pathway predominates, routing retinal afferents through the dorsolateral geniculate complex to the visual cortex. In most birds, the thalamofugal pathway often plays the lesser role with retinal afferents projecting to the principal optic thalami, a complex of several nuclei that resides in the dorsal thalamus. This thalamic complex sends projections to a forebrain structure called the Wulst, the terminus of the thalamofugal visual system. The thalamofugal pathway in birds serves many functions such as pattern discrimination, spatial memory, and navigation/migration. A comprehensive analysis of avian species has unveiled diverse subdivisions within the thalamic and forebrain structures, contingent on species, age, and techniques utilized. In this study, we documented the thalamofugal system in three dimensions by integrating histological and contrast-enhanced computed tomography imaging of the avian brain. Sections of two-week-old chick brains were cut in either coronal, sagittal, or horizontal planes and stained with Nissl and either Gallyas silver or Luxol Fast Blue. The thalamic principal optic complex and pallial Wulst were subdivided on the basis of cell and fiber density. Additionally, we utilized the technique of diffusible iodine-based contrast-enhanced computed tomography (diceCT) on a 5-week-old chick brain, and right eyeball. By merging diceCT data, stained histological sections, and information from the existing literature, a comprehensive three-dimensional model of the avian thalamofugal pathway was constructed. The use of a 3D model provides a clearer understanding of the structural and spatial organization of the thalamofugal system. The ability to integrate histochemical sections with diceCT 3D modeling is critical to better understanding the anatomical and physiologic organization of complex pathways such as the thalamofugal visual system.

## Introduction

The neuroanatomical pathways governing vision in vertebrates are diverse and functionally specialized^1,2^. These systems are critical for the development, reproduction, and survival of most vertebrates, making them an important aspect of neurobiological research. Avian species often exhibit advanced visual capabilities that are comparable to or exceed those of other vertebrates and are crucial for complex behaviors such as predator detection, short and long-distance migration, mating, and foraging and predation^3,4^. This makes avian species excellent model organisms for studying the pathways of vision.

Most of our current knowledge of the avian visual systems have been revealed using conventional approaches, such as gross dissection or 2D histology. These approaches have yielded much information previously unknown within avian neuroscience including characterization of photoreceptor diversity among species^5^, reciprocal columnar organization in the forebrain^6^, and unique anatomical subdivisions of various visual structures^7^. For instance, the thalamofugal system—a sequence of circuits important to visual processing—has been thoroughly described in the pigeon^8–10^, chicken^11–14^, owl^8,15,16^ and to some degree the zebra finch^17,18^ in terms of its intricate anatomy and to a lesser extent, functionality. The thalamofugal pathway consists of two primary neuronal projections. The first originates from retinal ganglion cells (RGCs) whose axons form the optic nerve and mostly decussate to the contralateral brain where the axons curve into the thalamus to reach nuclei of the dorsal lateral geniculate nuclear complex (GLd)^19^. The second projection arises from differing cell populations of the GLd that project ipsilaterally, contralaterally, or bilaterally to the Wulst^11,12,20,21^. The Wulst can be divided into at least four regions, depending on cell morphology, cell density, connectivity, and differential staining^8,13,22^ (see below for further discussion of neuroanatomical subunits). The Wulst represents the terminus of the thalamofugal circuit; however, it has several intratelencephalic projections to higher visual associative regions that enable more complex processing and spatial memory formation^14,23,24^. Additionally, the Wulst has several extratelencephalic targets such as the optic tectum and nuclei within the GLd likely performing top-down modulation^23,25^.

Notably, these investigations utilized traditional 2D imaging techniques to study the anatomy of the thalamofugal system. In an attempt to capture 3D relationships, 2D images were often aligned in series to document this pathway in 3D. However, this method has challenges including that it necessarily produces an incomplete 3D visualization, usually due to section alignment issues, sample damage during histological preparation, and relatively larger inter-slice spacing. These challenges can result in an incomplete understanding of delicate neuroanatomical features and an underappreciation of the neuroanatomical complexity of the system. Thus, the demand arises for improved 3D visualization techniques that are especially important for documenting highly integrated neural circuits within the brain. For example, the ability to investigate avian neuroanatomy in 3D should enable a more detailed understanding of neural systems and allow for a deeper appreciation of spatial complexity, structural relationships, brain region variation, and developmental mechanisms that define them^26–28^.

With recent developments in imaging techniques, we now have an opportunity to fill this gap. One such technique, diffusible iodine-based contrast-enhanced computed tomography^29^ (diceCT), offers an effective mode of visualizing vertebrate nervous tissue in 3D and at relatively high resolution. DiceCT enhances visualization of neural regions by utilizing contrast agents that selectively bind to white and grey matter, and subsequently produces brain datasets with tissue contrast at resolutions comparable to micro-MRI^30,31^. Additionally, diceCT can be paired with traditional histological datasets to generate multi-scale levels of detail from cell to whole organism^32,33^. Together, this provides the capability for developing highly detailed, comprehensive, 3D model visualizations of the thalamofugal pathway.

Here we aim to use diceCT to create a current, comprehensive, and interactive 3D brain model, illustrating detailed thalamofugal structures utilizing the chick (*Gallus gallus*) as a model. Our model features in-depth structural data and spatial organization that delineates the flow of information within this nested, recursive system^7,11,12,19–21^. To provide a detailed model of the thalamofugal system for future use in avian neurobiological research and education, we utilize a combination of serial stacked histochemical brain sections and diceCT for 3D reconstruction of visual components of the avian brain to produce a comprehensive anatomical description of the thalamofugal pathway. To achieve this, we met three objectives: (1) digitally reconstruct the thalamofugal nuclei of the dorsolateral geniculate complex, (2) digitally reconstruct the visual Wulst and other forebrain areas implicated in visual function within the thalamofugal system, and (3) display the interconnectivity among these structures. We anticipate that the 3D biomodel of the thalamofugal pathway presented herein will not only catalyze further research into the avian visual system, but also pave the way for more integrative insights into the neural systems within the avian brain, fostering advancements in our scientific understanding of avian neural complexity.

## Results

We describe the neuroanatomy of the chicken thalamofugal system by following the direction of information flow, starting at the retina. The circuit is composed of two primary neuron projections. The first begins with the retinal ganglion cells (RGCs) whose cell bodies reside in the ganglion cell layer of the retina. The RGCs receive input from bipolar neurons and send projections out of the back of the eye as the optic nerve. Most of the axons in each optic nerve next decussate at the optic chiasm, which allows them to enter the contralateral diencephalon. Here the axon terminals synapse in various visual nuclei that make up the dorsal lateral geniculate (GLd) complex. These nuclei produce the second primary neuronal projections that send ipsilateral, contralateral, or bilateral input to the visual Wulst in the dorsomedial forebrain (see supplementary figure S1).

### Retina

Processing of visual information begins at the level of the retina, a multilayered structure with several unique sets of cells that reside within various sublaminae. Visual field properties are processed by several classes of photoreceptors that send these signals to RGCs via bipolar cell mediators (e.g., chicken^5,34,35^, pigeon^36^, zebra finch^37^). Retinal ganglion cells primarily from the yellow field, the large central region of the retina, project through the optic nerve to the contralateral dorsal geniculate nuclear complex via the optic tract^19,38^. Each nuclear component of the GLd (see next section) receives a different distribution of contralateral visual input.

**Figure 1:**
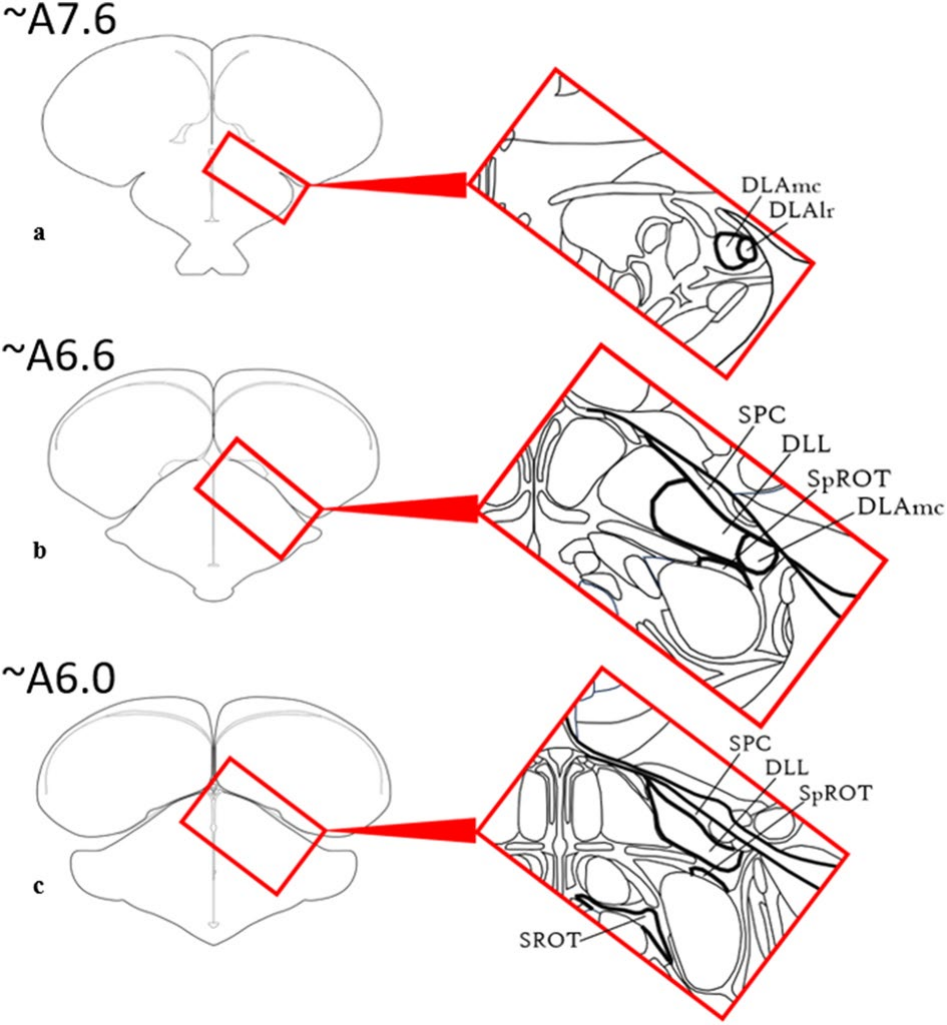
Location of thalamofugal nuclei in the thalamus. Series of redrawn rostral to caudal coronal sections (**a-c**) describing the complexity of the thalamus and outlining (in bold) the important nuclei involved in the thalamofugal system. Part **a** demonstrates the DLAmc and DLAlr in the anterior thalamus. Part **b** demonstrates the SPC, DLL, SpROT, and DLAmc in the intermediate thalamus. Part **c** demonstrates the SPC, DLL, SpROT, and SROT in the caudal thalamus.

**Figure 2:**
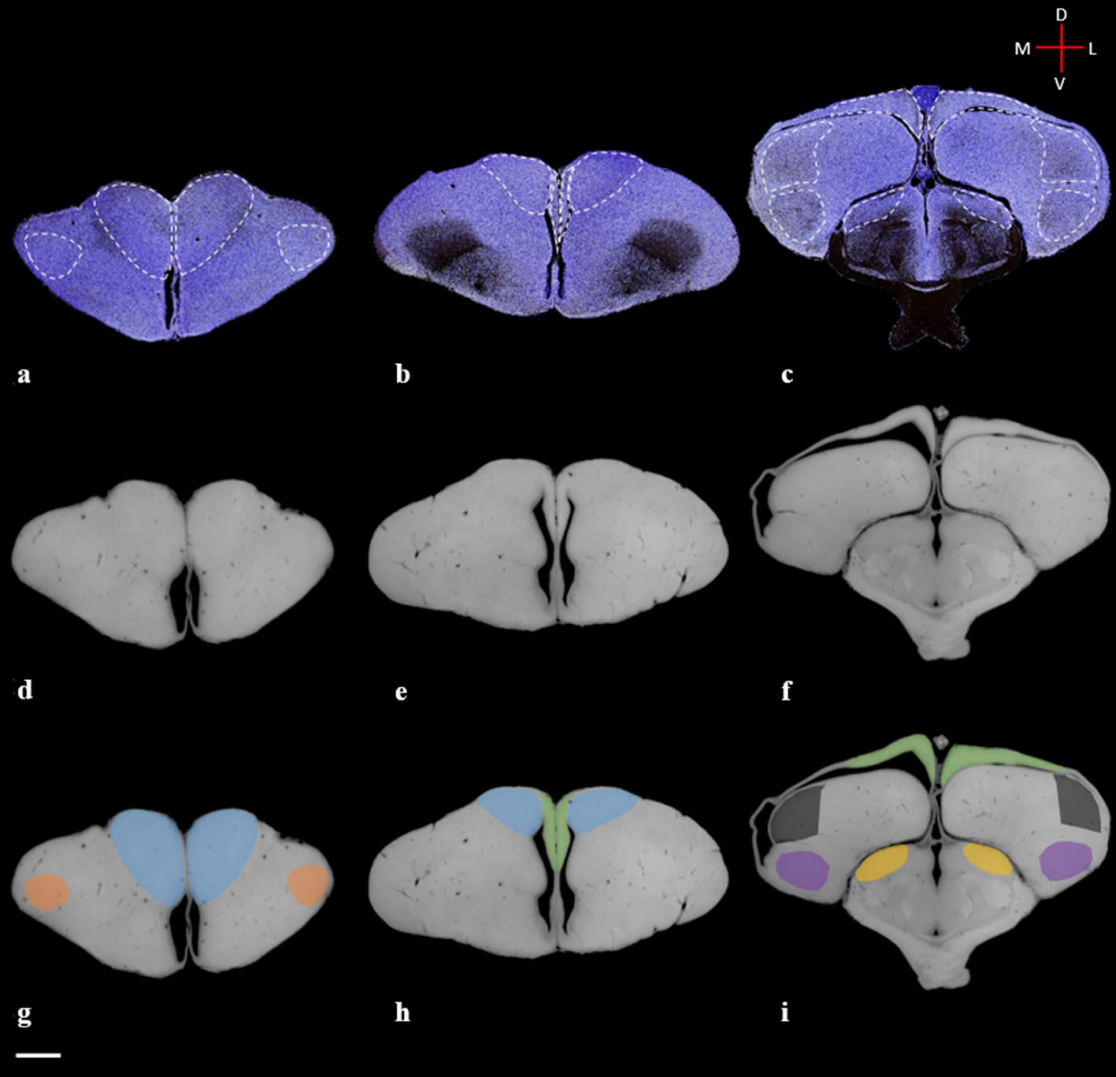
Series of rostral-to-caudal coronal brain sections in Gallyas silver stain with dashed outlines of thalamofugal structures (**a–c**) and diceCT (**d–i**) visualization protocols with various thalamofugal structures highlighted showing similar features in both preparation methods. **g,h,i:** diceCT scanned sections with overlay of thalamofugal structures (blue: Wulst; green: hippocampal area; gray: caudolateral nidopallium; orange: frontolateral nidopallium; purple: arcopallium). Scale bar = 2 mm.

**Figure 3:**
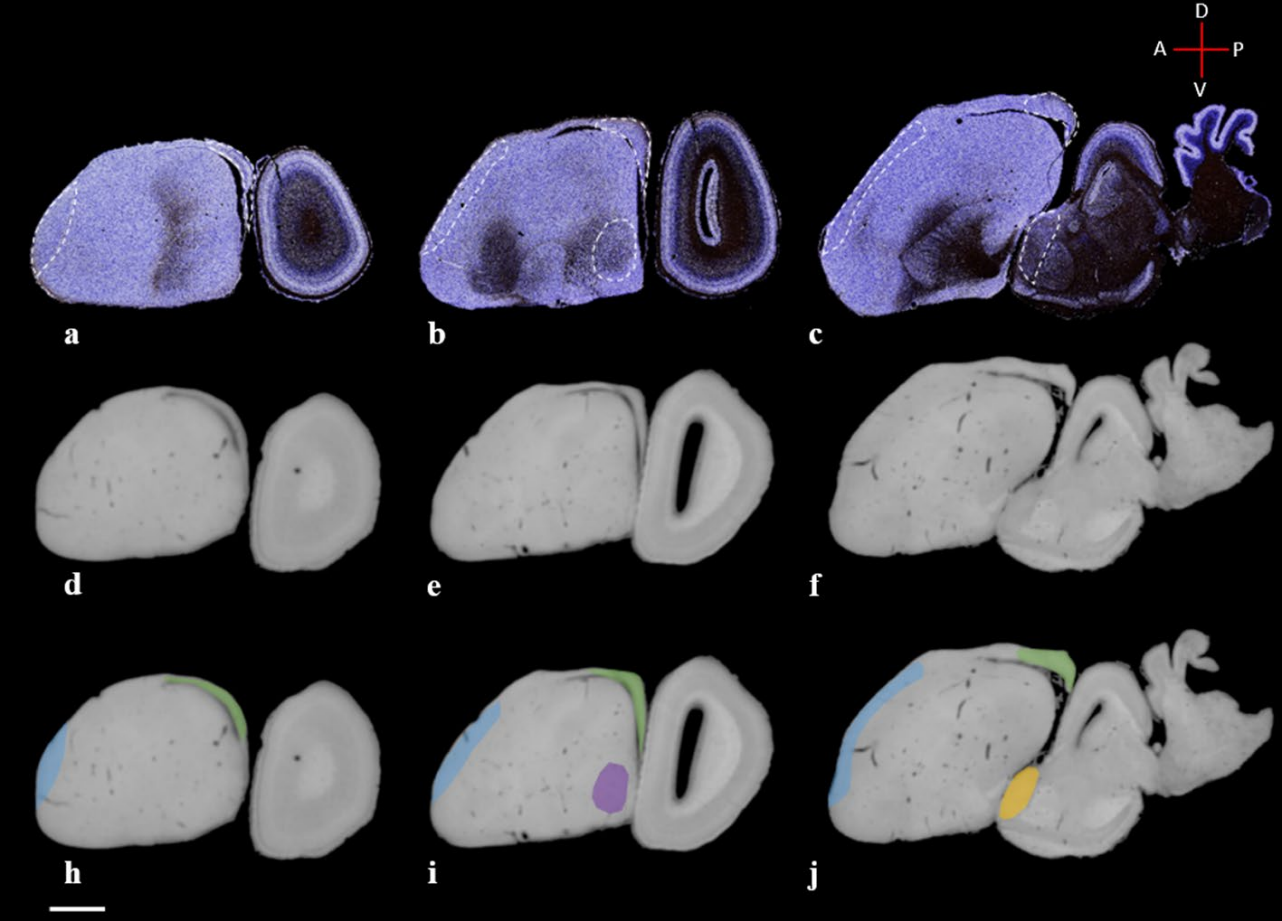
Series of lateral-to-medial sagittal brain sections in Gallyas silver stain with dashed outlines of thalamofugal structures (**a–c**) and diceCT (**d–i**) visualization protocols showing similar features in both preparations. **g,h,i:** diceCT scanned sections with overlay of thalamofugal structures (blue: Wulst; green: hippocampal area; purple: arcopallium; yellow: GLd). Scale bar = 2 mm.

### Avian diencephalon, thalamus

#### DLAlr

**Figure 4:**
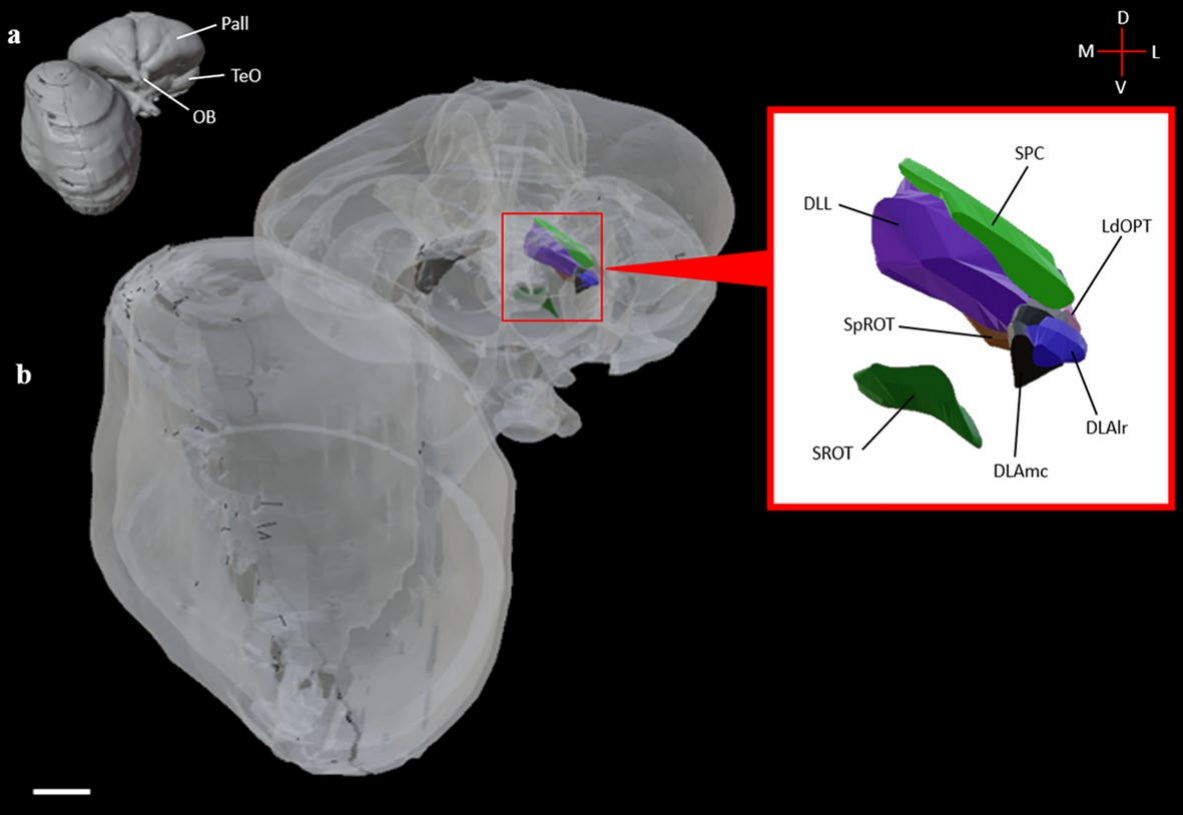
Frontal view of the diceCT eyeball and brain. **a:** Structures rendered as fully opaque as a reference visualization. **b:** Rendered brain surface and eye have been made semitransparent to illustrate the dorsolateral geniculate complex as a whole (right hemisphere) and as its subdivided nuclear components (left hemisphere; DLAlr: dark blue; DLAmc: black; DLL: dark purple; LdOPT: light pink; SPC: light green; SpROT: brown; SROT: dark green). A callout box shows the thalamic components at a higher magnification. Labels: Pall, pallium; TeO, optic tectum; OB, olfactory bulb. Scale bar = 2mm. See supplementary table S1for structure clarification.

**Figure 5:**
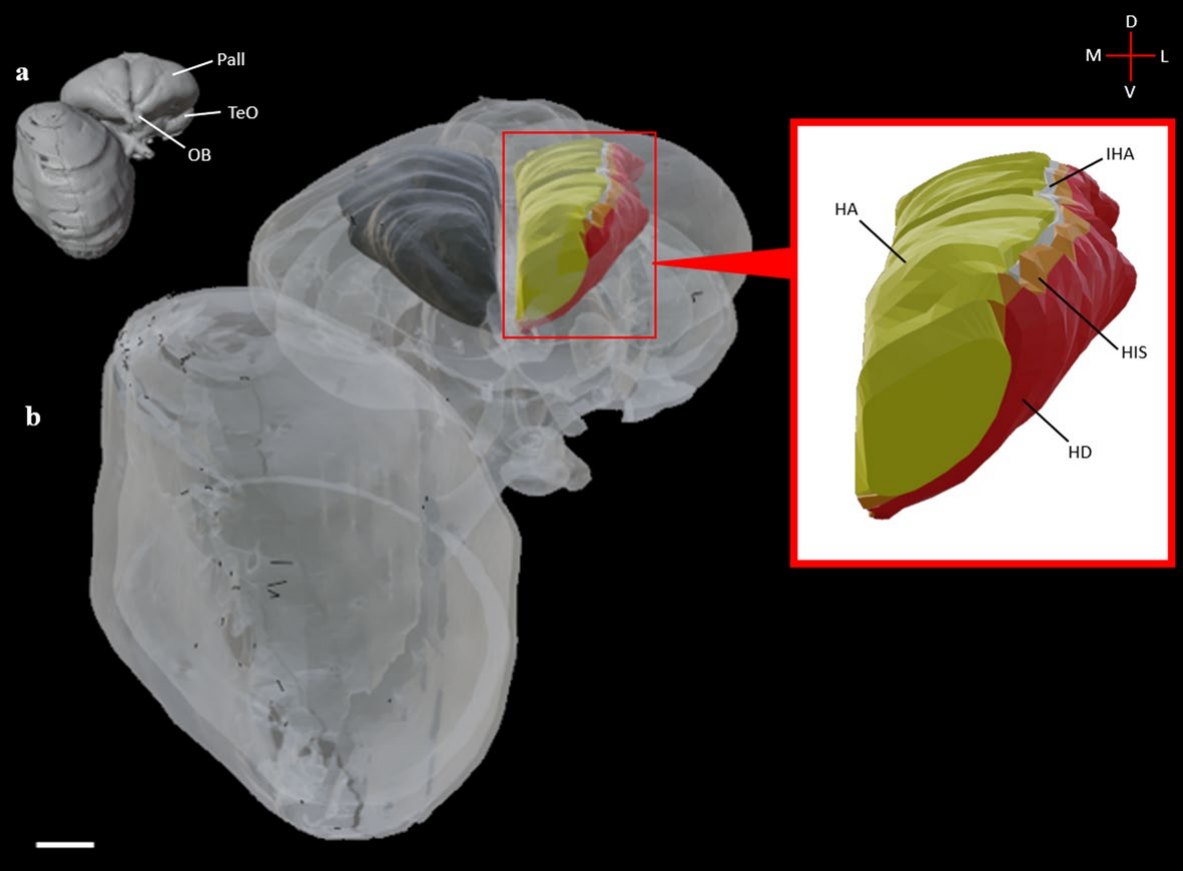
Frontal view of the diceCT eyeball and brain. **a:** Structures rendered as fully opaque as a reference visualization. **b:** Rendered brain surface and eye have been made semitransparent to illustrate the Wulst as a whole structure (right hemisphere) and subdivided components (left hemisphere; HD: red; HI: orange; IHA: white; HA: yellow). A callout box shows the Wulst components at a higher magnification. Labels: Pall, pallium; TeO, optic tectum; OB, olfactory bulb. Scale bar = 2mm. See supplementary table S1 for structure clarification.

**Figure 6:**
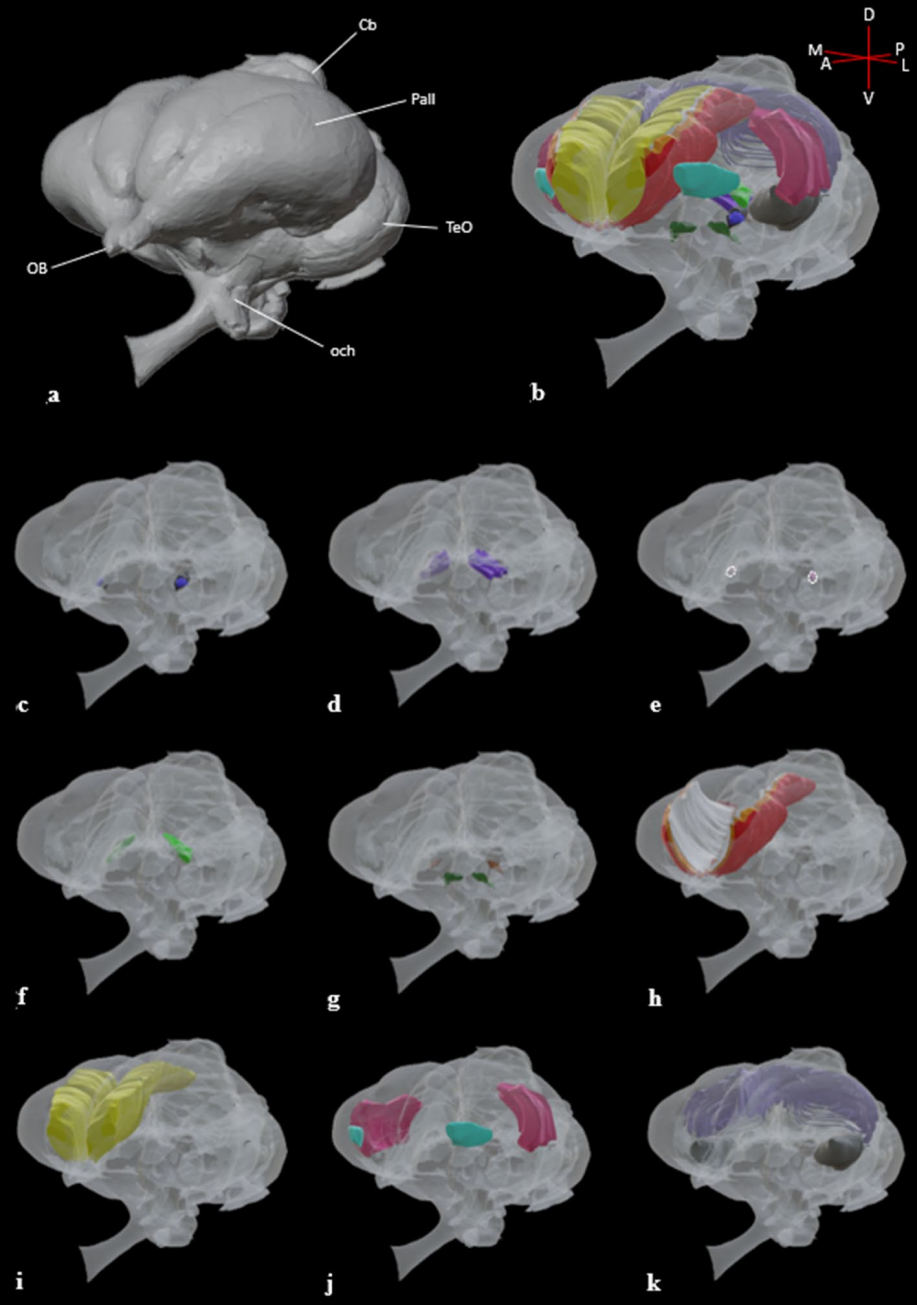
3D reconstruction of the thalamofugal nuclei. Structures are displayed in a semitransparent brain from a three-quarters left profile view. **a:** solid rendering of the diceCT brain as a reference visualization. **b:** semitransparent rendering of the diceCT brain with all the thalamofugal nuclei rendered opaque. **c:** DLAlr (dark blue) and DLAmc (black). **d:** DLL (dark purple). **e:** LdOPT (light pink with dotted white outline). **f:** SPC (light green) **g:** SpROT (brown) and SROT (dark green). **h:** HD (red), HI (orange) and IHA (white). **i:** HA (yellow). **j:** NFL (light blue) and NCL (dark pink). **k:** ARP (dark gray) and Hp (light purple). Labels: Pall, pallium; TeO, optic tectum; OB, olfactory bulb; Cb, cerebellum; och, optic chiasm. See supplementary tables S1 and S2 for structure clarification.

The dorsolateral anterior thalamus, lateral rostral part (DLAlr) is the most anterior nucleus of the GLd complex. The DLAlr receives comparatively weak retinal innervation^38^. DLAlr appears near atlas plate A8.0^39^ (Fig. 1a, 2c,f,I, 3) and is a small round nucleus that primarily projects to the contralateral Wulst. It was difficult to determine the boundaries of this nucleus toward its caudal end, but we were able to segment it and produce a 3D volume in Figs. 4, 5, 6c.

#### DLAmc

The dorsolateral anterior thalamus, magnocellular part (DLAmc) resides in the anterior half of the GLd, located caudally to the DLAlr. This structure maintains a round shape and first appears in coronal sections near atlas plate A 7.8^39^ (Fig. 1a, 2c,f,i). The DLAmc receives weak retinal innervation compared to other nuclei of the GLd complex. The DLAmc consists of mixed populations of cells that project either ipsilaterally using the lateral forebrain bundle, or contralaterally using the dorsal supraoptic decussation and then the lateral forebrain bundle. We reconstructed the DLAmc in Fig. 4 and 6c.

### DLL

The dorsolateral anterior thalamus, lateral part (DLL) is the largest component of the GLd complex. It first appears in coronal sections at plate A7.4^39^ and comprises the caudal portion of the GLd (Fig. 1b,c). This nucleus can be divided into ventromedial (DLLvm) and dorsolateral (DLLdl) subdivisions on the basis of cell density, differential projections, and choline acetyltransferase and glutamic acid decarboxylase staining^12,20,40,41^. The DLLdl is composed of mostly contralateral projections with minor ipsilateral projections to the Wulst, whereas the DLLvm is composed of mostly ipsilateral projections with minor contralateral projections to the Wulst^20^. Interestingly, recent studies in the garden warbler and homing pigeon have implicated this structure in magnetic compass orientation during night-time migration and navigation, respectively^42,43^. This recent evidence provides support for intricate subpathways that perform separate and distinct functions within the thalmofugal system. We were able to identify the DLL in both LFBS/CEV and GSM/N stains; however, subdivisions were only observable in the LFBS/CEV combination stain due to overwhelming staining of fibers in the GSM/N method (Figs. 4, 6d).

### SPC

The superficial parvocellular nucleus (SPC) is located dorsal and lateral to the DLL and maintains an elongated oval-like shape. The SPC receives weak retinal innervation from the contralateral retina and projects primarily to the contralateral Wulst^20,44^. The SPC can be divided into dorsal and ventral subdivisions by using cell size as the dorsal subdivision is characterized by small cells and the ventral division characterized by medium-sized cells^41^. Unfortunately, we could not make out this demarcation likely due to the passing fibers of the corticoseptomesencephalic (csm) tract that runs through the SPC. The SPC was distinguishable in both sets of coronal series as an oval-like structure with an elongated tail passing medially (Figs. 1b,c, 4, 6f).

#### SpROT

The suprarotundus (SpROT) is a flat nucleus that resides ventral to the DLL and covers the dorsal anterior nucleus rotundus. This structure is distinct from the dorsal DLL and rotundus located ventrally because of its darkly stained and tightly packed cells. The SpROT can also be differentiated from surrounding structures based on glutamic acid decarboxylase and neuropeptide Y staining^41^. The SpROT sends strictly ipsilateral projections to the Wulst utilizing the lateral forebrain bundle. Due to the easily identifiable nature of the SpROT, we were able to segment and produce a volume for its structure (Figs. 1b,c, 4, 6g).

#### SROT

The subrotundus nucleus (SROT) lies ventromedial to the rotundus in the thalamus and is triangular in shape. Like the SpROT, the SROT is easily discernible because of its dark and densely packed cells. SROT does not seem to receive direct retinal input, however, does send notable ipsilateral projections to the Wulst^20,38^. Interestingly, the SROT may also directly project to a portion of the hippocampal formation^45^. Because the SROT is easily visible, we were able to segment and reconstruct its structural volume from our datasets (Figs. 1c, 4 and 6g).

#### LdOPT

The lateral dorsal principal optic thalamus (LdOPT), also known as the anterodorsal nucleus, sits ventral to the SPC and lateral to the DLL. This is the smallest nucleus of the GLd that maintains a round to oval shape through its rostrocaudal extent. The LdOPT receives dense input from the contralateral retina and projects onto the contralateral Wulst^12,38,44^. This small nucleus was difficult to determine within our coronal histological series, so we utilized several descriptions from the literature^9,12,44^ to aid in the segmentation of this structure in Fig. 4 and 6e.

### Avian telencephalon, pallium

#### Wulst

The Wulst is the terminal structure of the thalamofugal pathway and occupies a large extent of the dorsomedial pallium. This structure is multilayered, presenting at least 4 regions (HA, apical hyperpallium; IHA, intercalated nucleus of the apical hyperpallium; HI, intermediate hyperpallium; and HD, densocellular hyperpallium) and is often described as the homolog of the striate cortex^46^ (see supplementary figure S2). The visual Wulst receives bilateral projections from the nuclei of the GLd and projects to several intratelencephalic regions (NCL, NFL, ARP, Hp) and extratelencephic regions^13,23^ (GLd, TeO). Several functional studies have shown involvement of the Wulst in numerous complex functions such as arena pattern discrimination^47^, color reversal learning, sun compass orientation^48^, and categorization of food versus nonfood^44^. The Wulst occupies much of the dorsomedial brain surface and can be delineated at its ventral aspect using the dorsal mesopallial lamina and to some degree the medial vallecula (a vascular groove in the forebrain along the lateral margin of the Wulst). We segmented the Wulst and delineated the HA, HI, IHA and HD subdivisions (Figs. 3a-c, 5, 6h-i).

#### Nidopallium

The nidopallium, the region of the forebrain residing between the ventral mesopallial lamina and subpallial lamina, consists of four visual associated regions: the intermediate nidopallium (NI), the frontal lateral nidopallium (NFL), the intermediate lateral nidopallium (NIL), and the caudal lateral nidopallium (NCL). Although each of these regions have been implicated in visual function^4,6,23,49–51^, only the NCL and NFL have been shown to be directly involved in the thalamofugal system.

The NFL lies rostral and lateral to the entopallium and maintains a spherical shape. The NFL has been shown to have reciprocal projections to the Wulst^13^ as well as sending projections to the NIL and intermediate arcopallium^13,23^. The NFL has been shown to be involved in context coding and extinction learning^49^. We segmented the NFL through a series of GSM/N stained brain sections and generated its 3D volume shown in Fig. 6j.

The caudal portion of the visual nidopallium (NCL) sits behind the NIL and has reciprocal connections with the Wulst. The NCL has been suggested to be the homolog of the mammalian prefrontal cortex on the basis of dopaminergic innervation, connectivity, and regional multisensory convergence^50,52,53^. The NCL has been implicated in many visual functions such as reversal learning, color perception, and selection and execution of perceptual responses^50–52^. The NCL is often affiliated with the temporo-parieto-occipital (TPO) area as the border between the structures is difficult to define. For this reason, we likely include part or all of the TPO in our defined NCL boundaries (Fig. 6j).

#### Arcopallium

**Figure 7:**
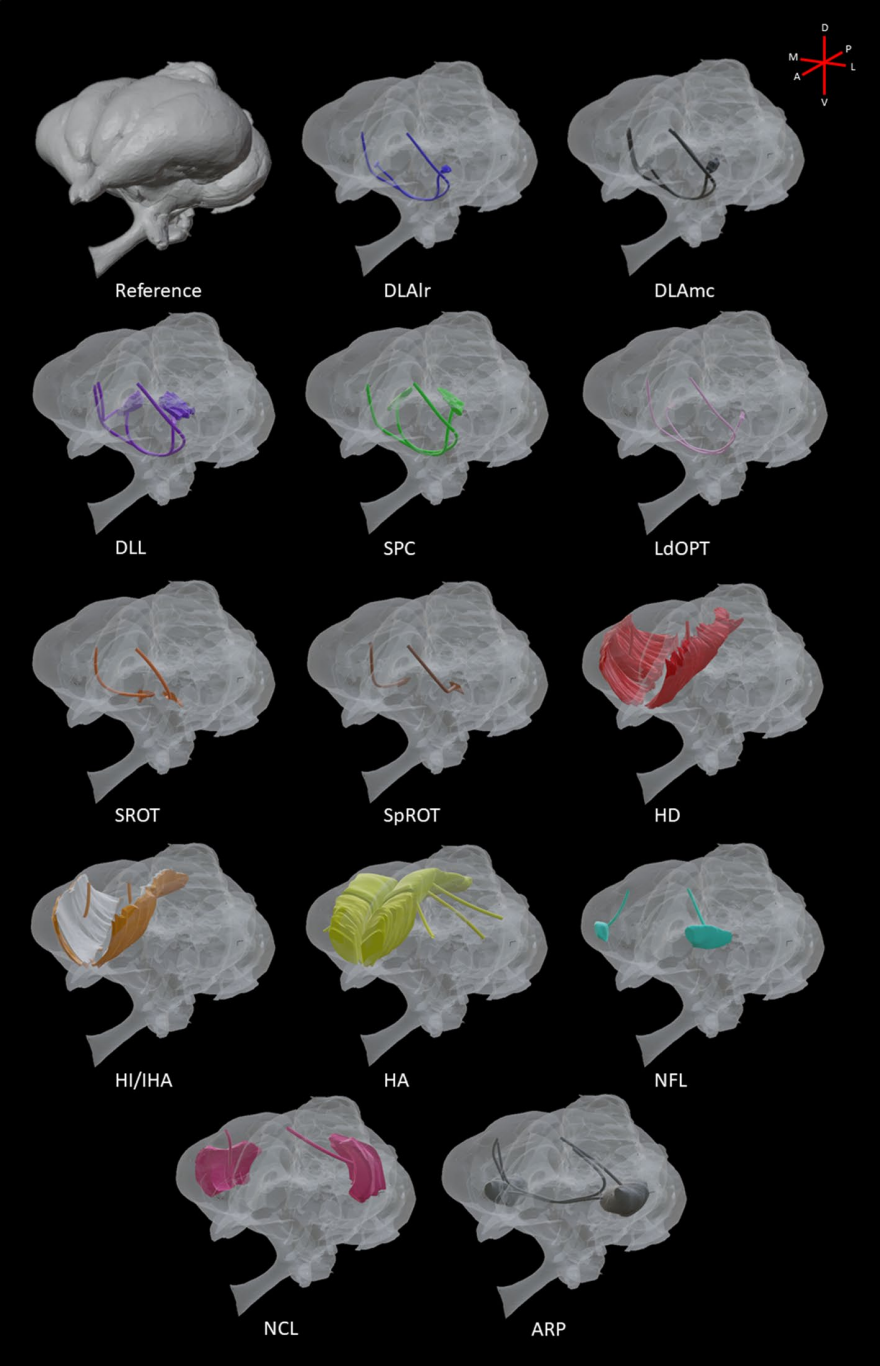
A three-quarters profile view illustrating 3D reconstruction of fiber tracts, decussations and commissures used by individual thalamofugal structures. Reference: shows the solid rendered brain. DLAlr: primarily projects to the contralateral Wulst using the dsd and lfb. DLAmc: projects bilaterally to the Wulst using the dsd and/or the lfb. DLL: projects bilaterally to the Wulst using the dsd and/or the lfb. SPC: primarily projects to the contralateral Wulst using the dsd and lfb. LdOPT: primarily projects to the contralateral Wulst using the dsd and lfb. SROT: primarily projects to the ipsilateral Wulst using the lfb. SpROT: primarily projects to the ipsilateral Wulst using the lfb. HD: projects up into the ipsilateral HA. HI/IHA: project up into the ipsilateral HA. HA: project to various ipsilateral pallial regions such as the ARP, NCL, Hp, and NFL. NCL and NFL: project onto the ipsilateral HA. ARP: projects bilaterally to the Wulst with contralateral projections utilizing the ac.

The arcopallium (ARP) can be divided into auditory, trigeminal, and visual sensory regions with the visual domain residing in the central area of the intermediate arcopallium (AI) and to some extent the dorsal arcopallium^54^ (AD). The visual arcopallium receives projections from the Wulst and sends reciprocal connections bilaterally back to the Wulst, utilizing the anterior commissure for contralateral projections^13,23,53^ (ac). The AI represents the beginning of the descending pathway that modulates visuomotor behaviors. The AI sends projections to the optic tectum, SPC, and SROT, likely modulating ascending visual input at the level of the tectofugal pathway and thalamofugal pathways^55^. For the purpose of delineating the thalamofugal system, we segment and display the AI and its connectivity in Figs. 6k and 7.

### Hippocampal area

The hippocampal formation (Hp) is an especially complex structure with varying subdivisions, often arranged depending on the methods used to investigate this region^17,45,56–58^. The Hp has reciprocal connections with the Wulst and is seemingly important for spatial memory and orientation making it a necessary component for behaviors such as navigation^59^, migration^60^, and food caching^61^. Though recent publications have noted specific divisions of the Hp to be involved in visually guided behaviors, for the purpose of this study we used our GSM/N (Fig. 3b-c) and diceCT (Fig. 3i-j) datasets to reconstruct the whole Hp rather than specific subdivisions(Fig. 6k).

### Fiber tracts, decussations and commissures

Based on the Gallyas silver fiber staining of coronal and sagittal sections, and intense labeling of myelinated fibers in the diceCT imaging, we reconstructed various fiber projections, tracts commissures, and decussations utilized by the thalamofugal system. Efferent and afferent connections of each structure were manually traced (with a few exceptions) and rendered in 3D. These renderings were replaced by uniformly smooth tubes that mimicked the natural paths axons would take to move from one structure to another. Fiber thickness was also manipulated in the model in an attempt to represent major and minor efferent and afferent projections (Fig. 7, supplementary figure S3).

## Discussion

**Figure 8:**
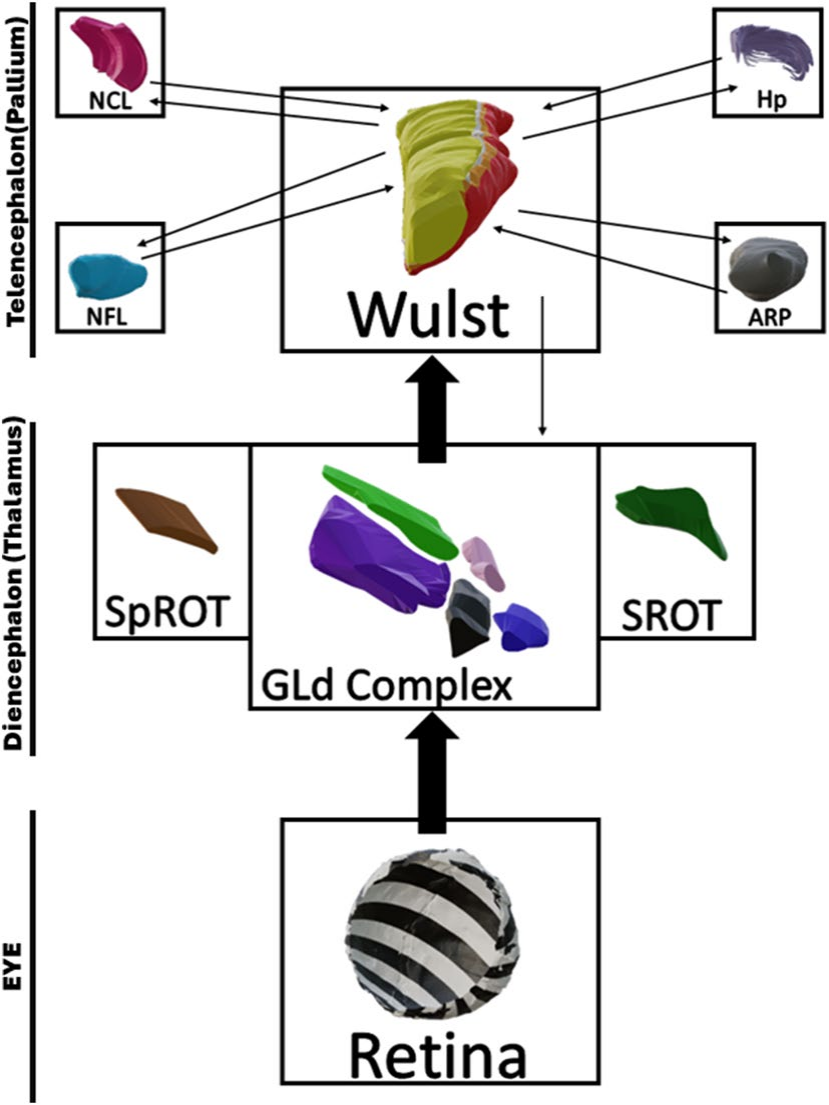
Summary of the major connections of the primary thalamofugal components (retina, nuclei of the GLd, Wulst) with the subsequent connections of the secondary thalamofugal components (NFL, NCL, ARP, Hp). Briefly, the nuclei of the GLd complex (DLAlr, DLAmc, DLL, LdOPT, SPC) receives projections from the retina. The complex of nuclei projects to three layers of the Wulst (HD, HI, IHA) that will subsequently project to the primary Wulst layer (HA). Additionally, two extra nuclei, SpROT and SROT, also project to the HD/HI/IHA. The HA is the source of intratelencephalic projections, projecting to the ARP, NFL, NCL and Hp. The HA also contains extratelencephalic projections to the optic tectum and various nuclei of the GLd. The NFL and NCL project back to the Wulst. ARP sends projections bilaterally to the Wulst.

In the present study, we utilized diceCT and a series of histological brain sections to reconstruct in 3D a highly detailed, comprehensive model of the chicken thalamofugal pathway. We identified and bilaterally reconstructed 12 thalamofugal structures, subdivided the Wulst into four divisions, and illustrated the many tracts, commissures, and decussations utilized by this visual pathway (see Fig. 8 for a summary). By integrating diceCT, with traditional histochemistry, we illustrate the thalamofugal system in 3D, featuring cellular and gross neuroanatomical phenotypes and their interconnections.

Traditional histology has been and continues to be a fundamental part of understanding neural development, anatomical organization, and connectivity. This technique has proven vital for understanding the embryonic neural development^9^, intricate neuronal connectivity^14,17,45,52^, identification of different neuronal types^14,22,41^, and assessing variable brain region functionality^10,62,63^. We utilized traditional histology in our study to support detailed two-dimensional and three-dimensional reconstructions of various thalamofugal structures and fiber systems. The histological brain sections allowed for better isolation of neural structures and fiber tracts utilized by the thalamofugal visual system. Additionally, these 2D sections assisted in the accurate placement of reconstructed thalamofugal nuclei and fiber systems within the 3D rendered whole brain volume. Histology provides higher resolution, cellular and subcellular detail for determining, and the means for tissue and cell morphological classification that is typically unobtainable by computed tomography.

DiceCT has become a popular method for investigating vertebrate anatomy. Several recent avian anatomical studies have utilized diceCT to gain insight into the feeding apparatus^64,65^, vocal organs^66^, craniofacial pathology^32^, cranial musculoskeletal anatomy^67–70^, brain ontogeny and function^71,72^, and forelimb musculoskeletal anatomy^73^. The technique offers several attributes that traditional histochemistry often lacks, including low cost, ease of access, high precision imaging of 3D soft tissues, and reversibility of contrast enhancement (making it compatible with additional visualization tools like histology^32,33^). These attributes provide diceCT leverage for comparative neuroanatomical studies compared to conventional approaches. We implemented diceCT in our investigation to generate whole-brain and eye surface volumes as they would naturally exist in the skull. These generated models allowed for ready integration of thalamofugal structures for a comprehensive model of the thalamofugal system with accurate spatial organization, structural information, and connectivity. DiceCT also allows for easy shareability of data via volume surface files or as original stacks of TIFF images. This produces the opportunity for adaptation of these data as a foundation for future researchers to build upon.

Combining traditional histology with diceCT has enabled a novel channel for research. DiceCT allows users to obtain large-scale information about the whole brain and certain neural structures, while traditional histology allows for detailed sections of neural tissue for visualization of cell morphology and fiber tracts. Together, these different imaging techniques were used to create a 3D model of the thalamofugal pathway that is highly accurate in structural and spatial organization. Our model can be utilized in the understanding of the components of the thalamofugal pathway, its spatial organization, structure, and connectivity. For the purpose of research, our model will benefit anatomical investigations of the chicken thalamofugal pathway beyond the predefined orientations of conventional 2D imaging techniques. Specifically, this model can assist studies seeking to investigate a specific region of the thalamofugal pathway using electrophysiology, intracellular filling, and tract tracing. Additionally, diceCT enables virtual re-slicing at unorthodox angles for the purpose of visualizing as many structures as possible. DiceCT also has the capability to obtain volumetric data as the voxel size representing the shape and size of neural structures is known.

Generation of more thalamofugal models in the chicken would benefit 3D investigation of the ontogenesis of this pathway as well as the generation of thalamofugal models in other avian species to compare interspecific differences and possibly ascertain functional implications. We anticipate that these comparisons will expand our understanding of the alterations of structural volume, spatial organization, linear dimensions, and connectivity of components of the thalamofugal pathway and how these alterations are related to different visual ecologies of birds.

## Materials and methods

### Serial histochemical brain sections

All procedures utilized were approved by the University of Arkansas Institutional Animal Care and Use Committee and were carried out in accordance with relevant guidelines and regulations. Additionally, all procedures involving animals were performed in compliance with the ARRIVE guidelines (https://arriveguidelines.org). We used Luxol fast blue (LFBS), cresylecht violet (CEV), Gallyas Silver Myelin (GSM), and Nissl (N) staining of a two-week-old chicken brain for this study. LFB and CEV as well as GSM and N were paired for better identification of brain nuclei and fiber tracts. Luxol fast blue is a basophilic myelin sheath stain that colors nerve fibers blue as a major constituent of nerve processes is phospholipid. In contrast, CEV is an acidophilic agent that stains Nissl bodies (i.e., rough endoplasmic reticulum) dark purple. The GSM method is a silver stain that colors myelinated fibers (i.e., dendrites and axons) black. Pairing LFBS and CEV, and GSM and N together help to determine individual groups of cells defining a nucleus. GSM and N, and LFBS and CEV sets of slides were likewise available in three planes (Kuenzel, unpublished, histochemical sets of brain sections).

### Diffusible iodine-based contrast-enhanced computed tomography

Two birds at 5 weeks of age were completely anesthetized with sodium pentobarbital. Following anesthesia, a 5-week-old chick was perfused with 0.1 M phosphate-buffered saline, followed by 4% paraformaldehyde. After fixation, the sample was completely submerged in a 1% weight-by-volume (w/v) solution of iodine potassium-iodide (I_2_KI, a.k.a., Lugol's iodine) for 40 days at room temperature^29,31^. To promote staining, the calvarium and left lateral region of the braincase were partly removed and trepanned. This minimized the effect of a fully closed braincase ,which reduces stain diffusion rates, enabling usage of a relatively low (i.e., 1% w/v) concentration necessary for minimizing brain shrinkage^74^. When necessary, the solution was refreshed with an additional 1% I_2_KI, and this was performed to prevent the color of the Lugol's iodine staining from becoming paler over time.

The main aqueous form of Lugol's iodine (triiodide, I_3_^−^) has an affinity for sugars and lipids, and it naturally diffuses into the brain sample^29^. As the staining agent diffuses into the sample, the dark-colored I_3_^−^ polymers collect in areas with large amounts of sugars or lipids, such as the myelinated sheaths of oligodendrocytes and Schwann cells. Over time, the staining solution loses its dark appearance as the majority of I_3_^−^ becomes bound within the cells of the sample. This proves advantageous for X-ray imaging of brain tissue, because I_3_^−^ is radiopaque, allowing it to absorb X-rays during scanning. Consequently, it becomes feasible to distinguish white matter and other carbohydrate or lipid-rich tissue so that they are easily distinguished from neighboring structures. Collectively, this facilitates simultaneous 3D visualization of bony, nervous, muscular, epithelial, and specialized sensory structures.

### Image acquisition and processing

The diceCT sample was imaged using a 2018 Nikon XT H 225 ST µCT system (Nikon Metrology, Brighton, MI) at the MicroCT Imaging Consortium for Research and Outreach (MICRO) at the University of Arkansas, Fayetteville campus. The sample was sealed separately into 50 ml low-density, plastic tube filled with water (to prevent dehydration), and the tube was positioned within the scanning chamber for µCT imaging. To optimize scan parameters, a scout image was performed, following Gignac and Kley^31^. The sample was scanned at an isometric voxel size of 19.499 microns, using 211 kV, 91 µ-Amperage, a 708-ms exposure setting, and 8 × multi-frame averaging with a rotating tungsten target and 0.125 mm thick copper filter. The minimize ring artifacts setting was used to reduce the number of visible rings that may appear on scanned images. µCT volumes were reconstructed using Nikon µCT software and VG Studio Max (Volume Graphics GmbH, Heidelberg, Germany) on an HP z800 workstation (Hewlett‐ Packard, Palo Alto, CA, USA). The resulting dataset was exported as a stack of TIFF images for development of 3D models.

### Anatomical reconstruction

A combination of serial histochemical and diceCT datasets were used to reconstruct the whole brain surface and important structures of the thalamofugal pathway. To provide anatomical reference needed for the correct orientation of the whole brain and skull models, the inner ear canals, visible in the µCT scan, were rendered in 3D using AvizoLite 2020 (Thermo Fisher Scientific Inc., Waltham, MA). The reference orientation was consistent with the positioning of the brain and skull in a stereotaxic instrument and was used to standardize the positioning of histolochemical and diceCT data.

AvizoLite 2020 was used with the diceCT dataset to delineate the whole brain, including several thalamofugal structures. The whole brain surface and features of the thalamofugal system were 3D rendered from the background and surrounding tissues based on differences in grayscale contrast offered by differential iodine staining of white and gray matter^31,33^.

For segmentation in our histological datasets, semi-automatic and manual registration was performed for both the brain surface and thalamofugal structures, respectively. Segmentation of thalamofugal constituents was performed in coronal sections; however, sagittal and horizontal sections were available for reference. Serial histochemical sections were stacked after segmentation within Brainmaker (MBF Biosciences, Williston, VT) to produce 3D volumes of nuclear structures and fiber systems pertinent to the thalamofugal pathway. All 3D models from diceCT and serial histology were exported as surface files (.obj and .stl format). Files were imported into Blender (Blender Foundation, Amsterdam, Netherlands) and combined to create a complete representation of the thalamofugal visual system. Tables were created for figures displaying 3D elements (Figs. 4, 5, 6, 7) to summarize the structure name and color (see tables S1 and S2).

For spatial alignment of the datasets, stereotaxic images were imported into Blender. We first imported a midline sagittal atlas section and isometrically scaled it to match the brain surface in size and orientation. We then imported a series of coronal stereotaxic atlas sections and isometrically scaled them to align with the brain surface so that they crossed through the midline sagittal section at their respective atlas coordinates of Kuenzel and Masson^39^. 3D surface files were then manually aligned to the aforementioned spatial framework, integrating data from both diceCT and histology.

*Figure preparation.*

For Figs. 6 and 7, orientations of the brain and segmented thalamofugal components were chosen to maximize visualization. The Windows snipping tool software (version 10.2008.2277.0, Microsoft Corp., Redmont, WA, USA) was used to capture static images of the reconstructed 3D model. To enhance clarity, selected images were imported into GIMP 2.10.30 and the inherent grey background in Blender was removed and replaced with a transparent background. The smudge tool was used to fix mistakenly cropped information whose pixels had similar grayscale values to the background that was removed. The select and fill tools were then used to add a uniform black background to easily distinguish the semitransparent brain surface and solid neural structures and fibers underneath. For Figs. 2 and 3, histologic brain sections were selected due to their visualization of key thalamofugal structures and then paired to respective diceCT images. We chose to upsample diceCT images for the sole purpose of figure clarity, using TopazLabs (Dallas, TX, USA) Gigapixel AI image enhancement software. Upsampling these images involves subdividing existing pixels into several smaller subpixels where the Gigapixel AI machine learning algorithms interpret the grayscale values of each target pixel along with the surrounding pixels to interpolate a grayscale value for each new subpixel. This enables, for example, a square image that is 100 × 100 pixels to become a 600 × 600 pixel image, showing the same visualization, but with higher interpolated spatial detail. The use of thousands of image datasets to train Gigapixel AI machine learning algorithm also enables the software to scale noise, blur, and adjust luminance values to maintain image quality comparable to the original image. Essentially, this procedure enables magnification of 2D images beyond the original spatial resolution provided by the detector hardware and helical reconstruction method, while minimizing visual distortions.

### Terminology

For thalamofugal structures we primarily used the terminology from the atlas of Kuenzel and Masson^39^. For many of the forebrain regions, we adopted the nomenclature of Reiner et al. (2004)^75^. Furthermore, we used recent literature^76^ for nomenclature of several thalamofugal structures.

## Data availability

The datasets generated from this study are openly available on Figshare at [10.6084/m9.figshare.24883872] and [10.6084/m9.figshare.24883848]. Additional data are available from the corresponding author upon reasonable request.

### Supplementary Information

Supplementary Information 1.

Supplementary Information 2.

Supplementary Information 3.

Supplementary Information 4.

Supplementary Information 5.

Supplementary Information 6.

Supplementary Video 1.

Supplementary Video 2.

Supplementary Video 3.

## Abbreviations

ac: Anterior commissure
AD: Dorsal arcopallium
AI: Intermediate arcopallium
ARP: Arcopallium
CEV: Cresylecht violet
csm: Corticoseptomesencephalic tract
dat: Dorsal arcopallial tract
diceCT: Diffusible iodine-based contrast-enhanced computed tomography
DLAlr: Dorsolateral anterior thalamus, lateral rostral part
DLAmc: Dorsolateral anterior thalamus, magnocellular part
DLL: Dorsolateral anterior thalamus, lateral part
DLLd: Dorsal division of the dorsolateral anterior thalamus, lateral part
DLLv: Ventral division of the dorsolateral anterior thalamus, lateral part
dsd: Dorsal supraoptic decussation
lfb: Lateral forebrain bundle
GLd: Dorsolateral geniculate nuclear complex
GSM: Gallyas silver myelin
HA: Apical hyperpallium
HAI: Intercalated nucleus of the apical hyperpallium
HD: Densocellular hyperpallium
Hp: Hippocampal formation
HI: Intermediate hyperpallium
LdOPT: Lateral dorsal principal optic thalamus
LFBS: Luxol fast blue stain
N: Nissl
NCL: Caudolateral nidopallium
NFL: Frontolateral nidopallium
NI: Intermediate nidopallium
NIL: Intermediolateral nidopallium
OPT: Nucleus principal optic thalamus
RGCs: Retinal ganglion cells
SPC: Superficial parvocellular nucleus
SpROT: Suprarotundus
W: Wulst
