# Supplementary material for: A histological and diceCT-derived 3D reconstruction of the avian visual thalamofugal pathway
Source: Sci Rep. 2024 Apr 11;14:8447. doi: 10.1038/s41598-024-58788-z (PMC11006926; doi:10.1038/s41598-024-58788-z)
Supplement: Supplementary file 1 — Supplementary Information 1. [file 41598_2024_58788_MOESM1_ESM.docx]

Supplementary Figure Legends

Supplementary Figure S1. Schematic representation of the avian visual thalamofugal circuitry in the brain of the chicken. Specifically, this schematic shows the primary ascending connections, involving the retina, GLd (dorsolateral geniculate complex) and the Wulst.

Supplementary Figure S2. Series of coronal sections describing thalamofugal components. The series consists of 7 gallyas silver myelin and nissl stained coronal sections with boundaries of nuclei and their divisions shown with dotted white lines. Structures (nuclei and fibers) are labeled in red.

Supplementary Figure S3. Individual thalamofugal structures shown with their respective anterograde projections along with a reference image for spatial orientation.

Supplementary Table S1. Components on the dorsolateral geniculate complex. The table shows the thalamofugal structures within the thalamus and describes the structure’s full name, abbreviation and color within the 3D model.

Supplementary Table S2. Divisions of the thalamofugal Wulst. The table shows the thalamofugal structures within the pallium and describes the structure’s full name, abbreviation and color within the 3D model.

Supplementary Video S4. Coronal flythrough on the diceCT scan. Video flythrough of a series of digitally sliced coronal sections produced from our diceCT scan.

Supplementary Video S5. Sagittal flythrough of the diceCT scan. Video flythrough of a series of digitally sliced sagittal sections produced from our diceCT scan.

Supplementary Video S6. Horizontal flythrough of the diceCT scan. Video flythrough of a series of digitally sliced horizontal sections produced from our diceCT scan.
