# Supplementary figures and images for: A histological and diceCT-derived 3D reconstruction of the avian visual thalamofugal pathway
Source: Sci Rep. 2024 Apr 11;14:8447. doi: 10.1038/s41598-024-58788-z (PMC11006926; doi:10.1038/s41598-024-58788-z)

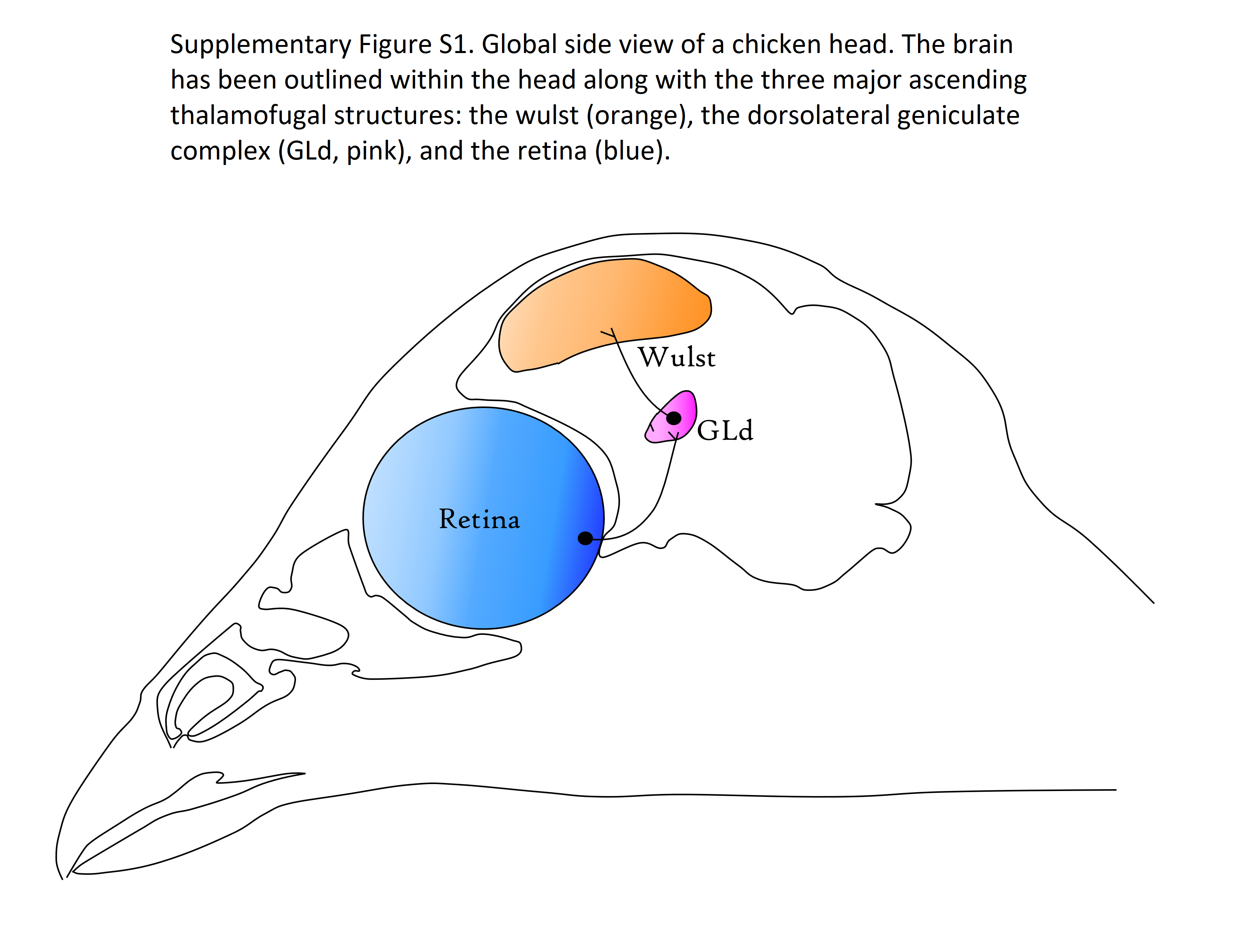

Supplement: Supplementary file 2 — Supplementary Information 2. [file 41598_2024_58788_MOESM2_ESM.png]

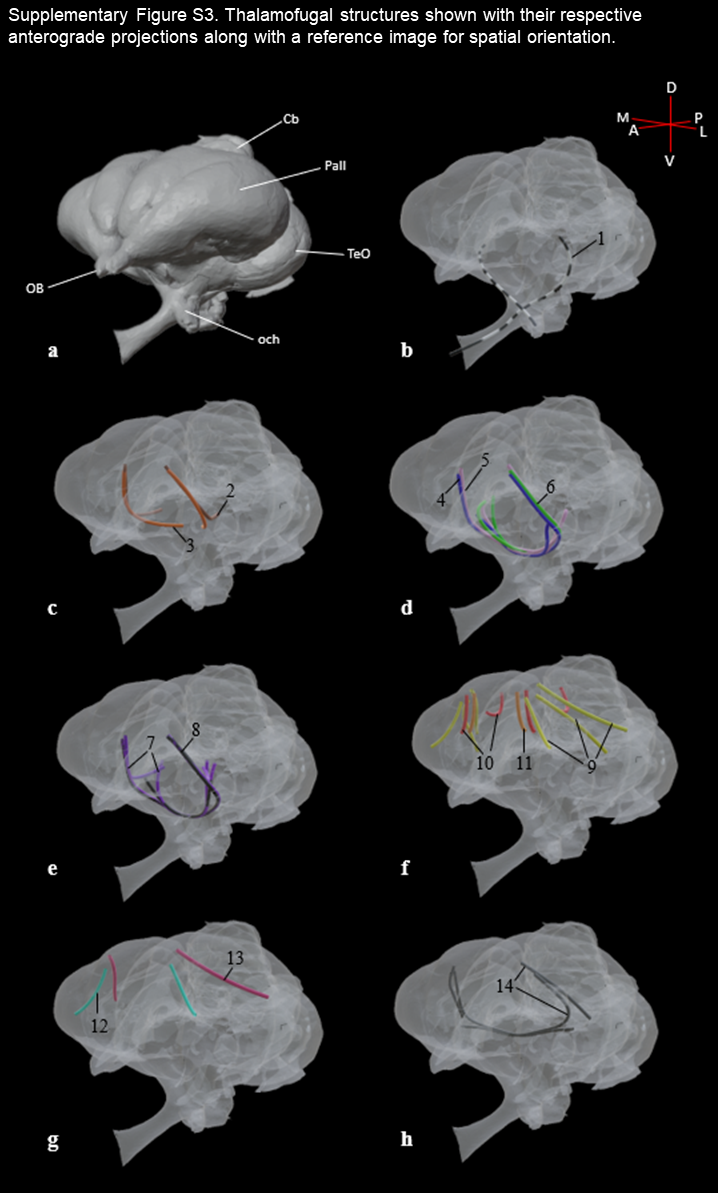

Supplement: Supplementary file 4 — Supplementary Information 4. [file 41598_2024_58788_MOESM4_ESM.tif]
