# Supplementary material for: A histological and diceCT-derived 3D reconstruction of the avian visual thalamofugal pathway
Source: Sci Rep. 2024 Apr 11;14:8447. doi: 10.1038/s41598-024-58788-z (PMC11006926; doi:10.1038/s41598-024-58788-z)
Supplement: Supplementary file 3 — Supplementary Information 3. [file 41598_2024_58788_MOESM3_ESM.pdf]

Supplementary Figure S2. A rostral to caudal series of Gallyas silver myelin and Nissl stained cross sections of a chick brain. Sections have important thalamofugal structures outlined in dotted white lines. Structures and fiber tracts are highlighted with red lines/labels. Abbreviation legends have been provided within each section.

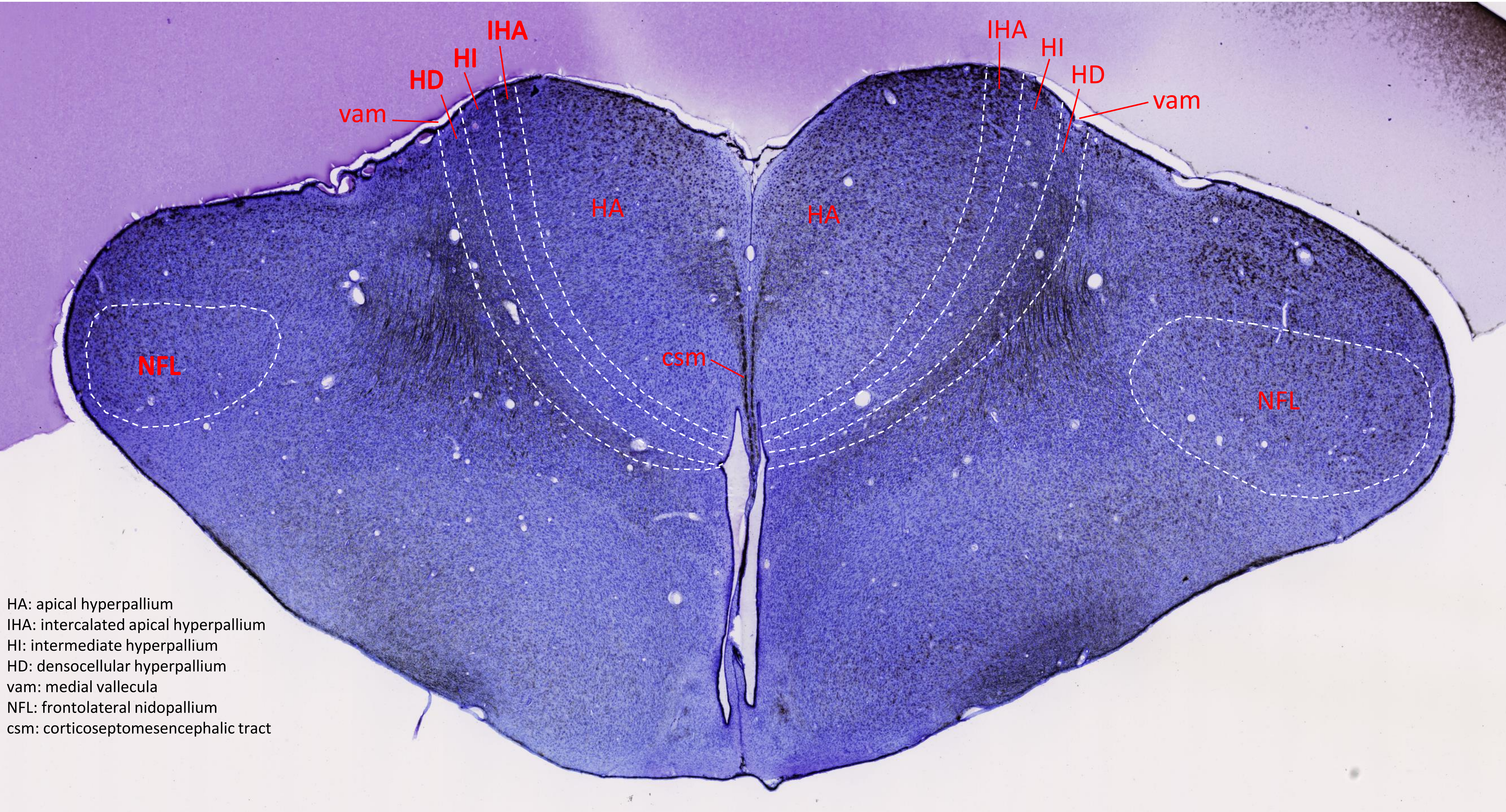

HA: apical hyperpallium
IHA: intercalated apical hyperpallium
HI: intermediate hyperpallium
HD: densocellular hyperpallium
vam: medial vallecula
NFL: frontolateral nidopallium
csm: corticoseptomesencephalic tract

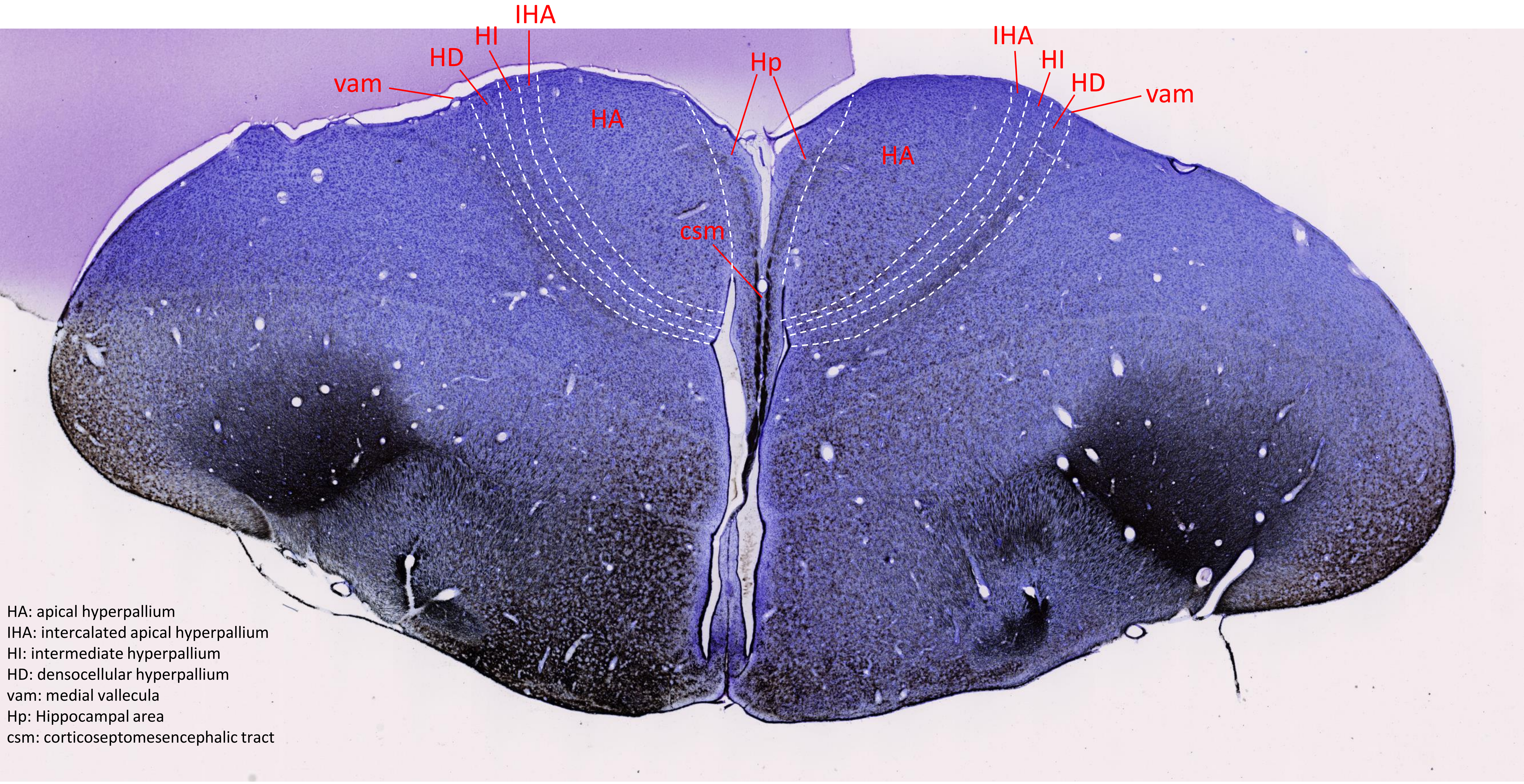
IHA
HI
HD
vam
Hp
HA
csm
IHA
HI
HD
vam
HA
HA: apical hyperpallium
IHA: intercalated apical hyperpallium
HI: intermediate hyperpallium
HD: densocellular hyperpallium
vam: medial vallecula
Hp: Hippocampal area
csm: corticoseptomesencephalic tract

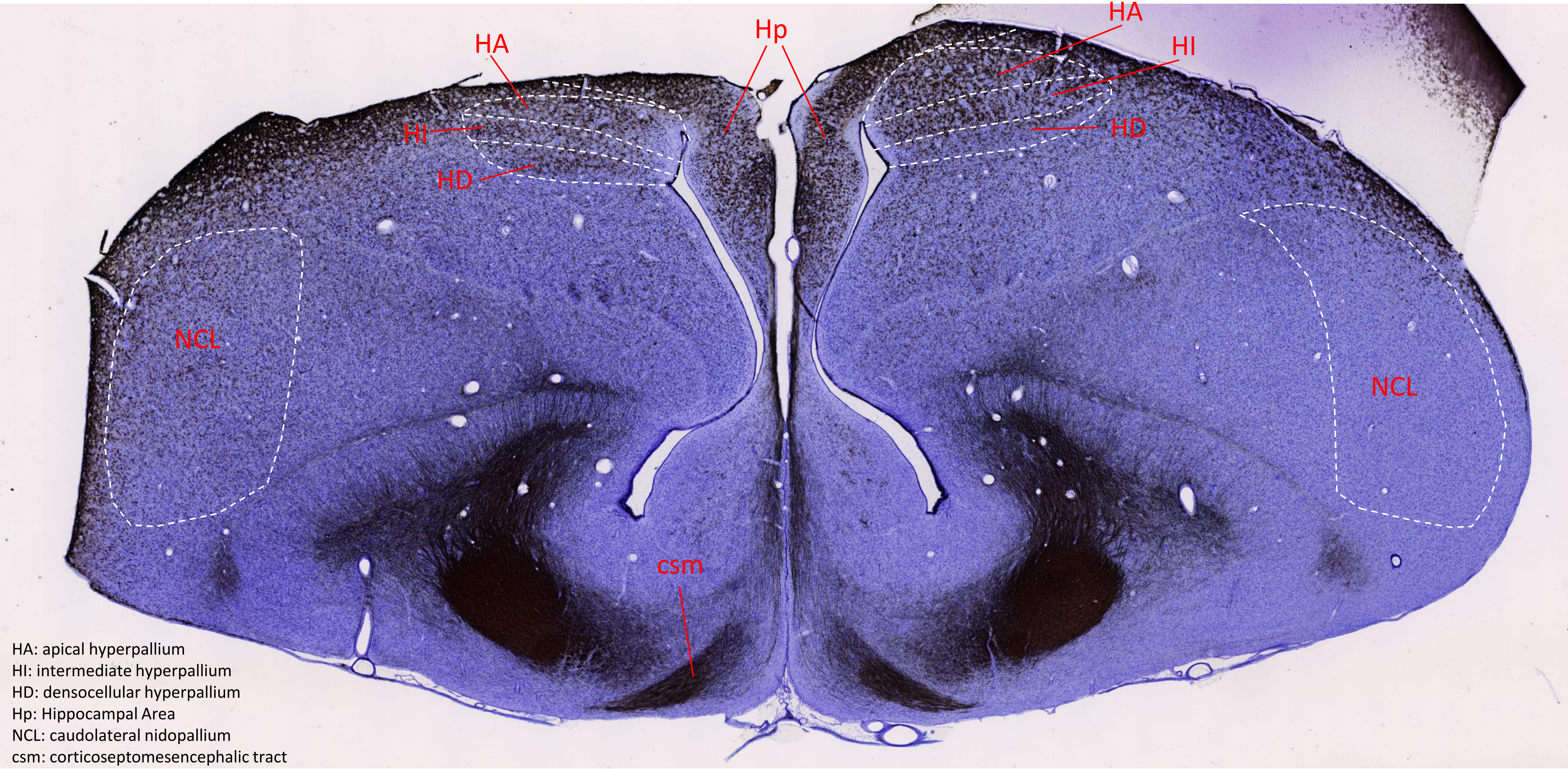

HA: apical hyperpallium
HI: intermediate hyperpallium
HD: densocellular hyperpallium
Hp: Hippocampal Area
NCL: caudolateral nidopallium
csm: corticoseptomesencephalic tract

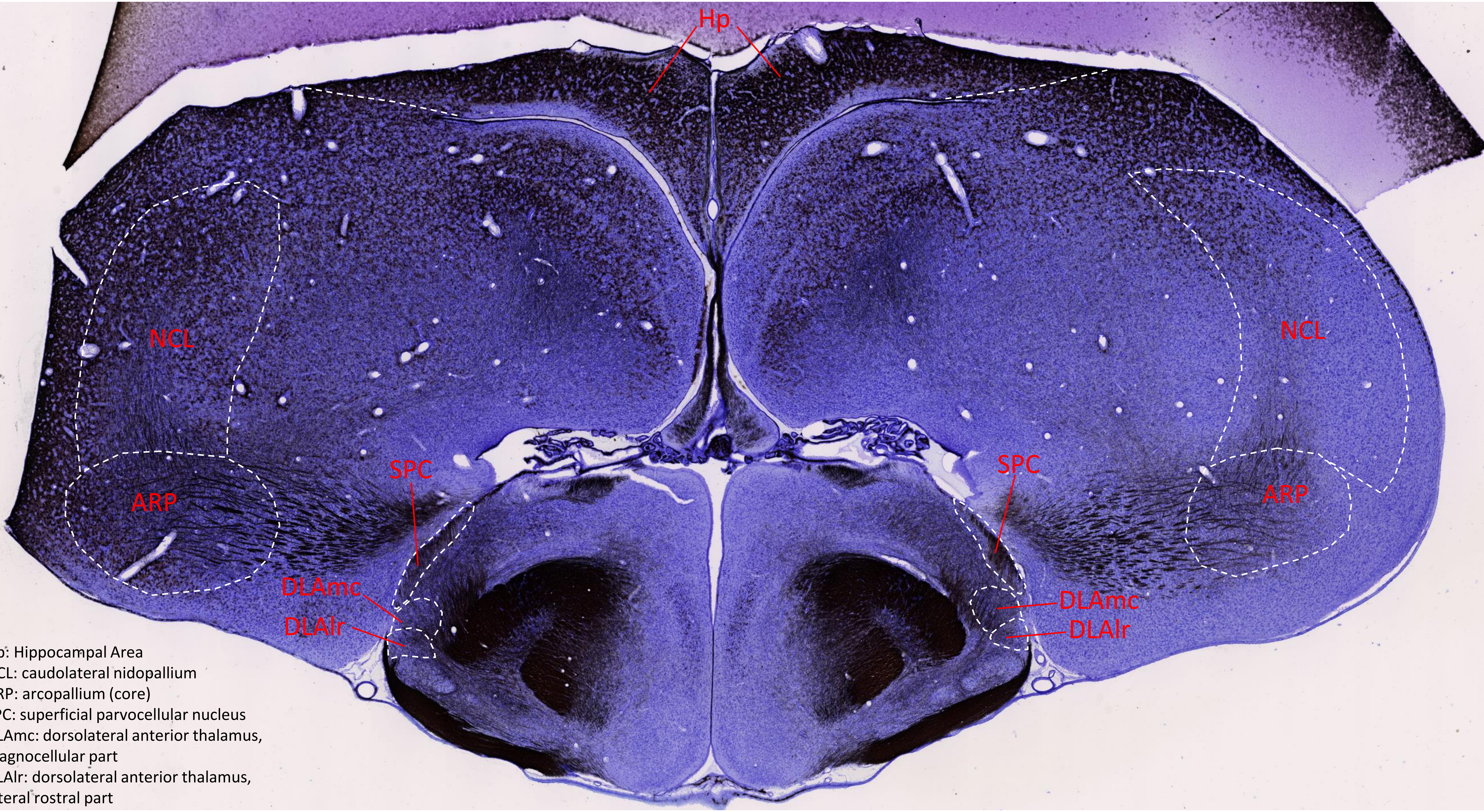

Hp: Hippocampal Area
NCL: caudolateral nidopallium
ARP: arcopallium (core)
SPC: superficial parvocellular nucleus
DLAmc: dorsolateral anterior thalamus, magnocellular part
DLAlr: dorsolateral anterior thalamus, lateral rostral part

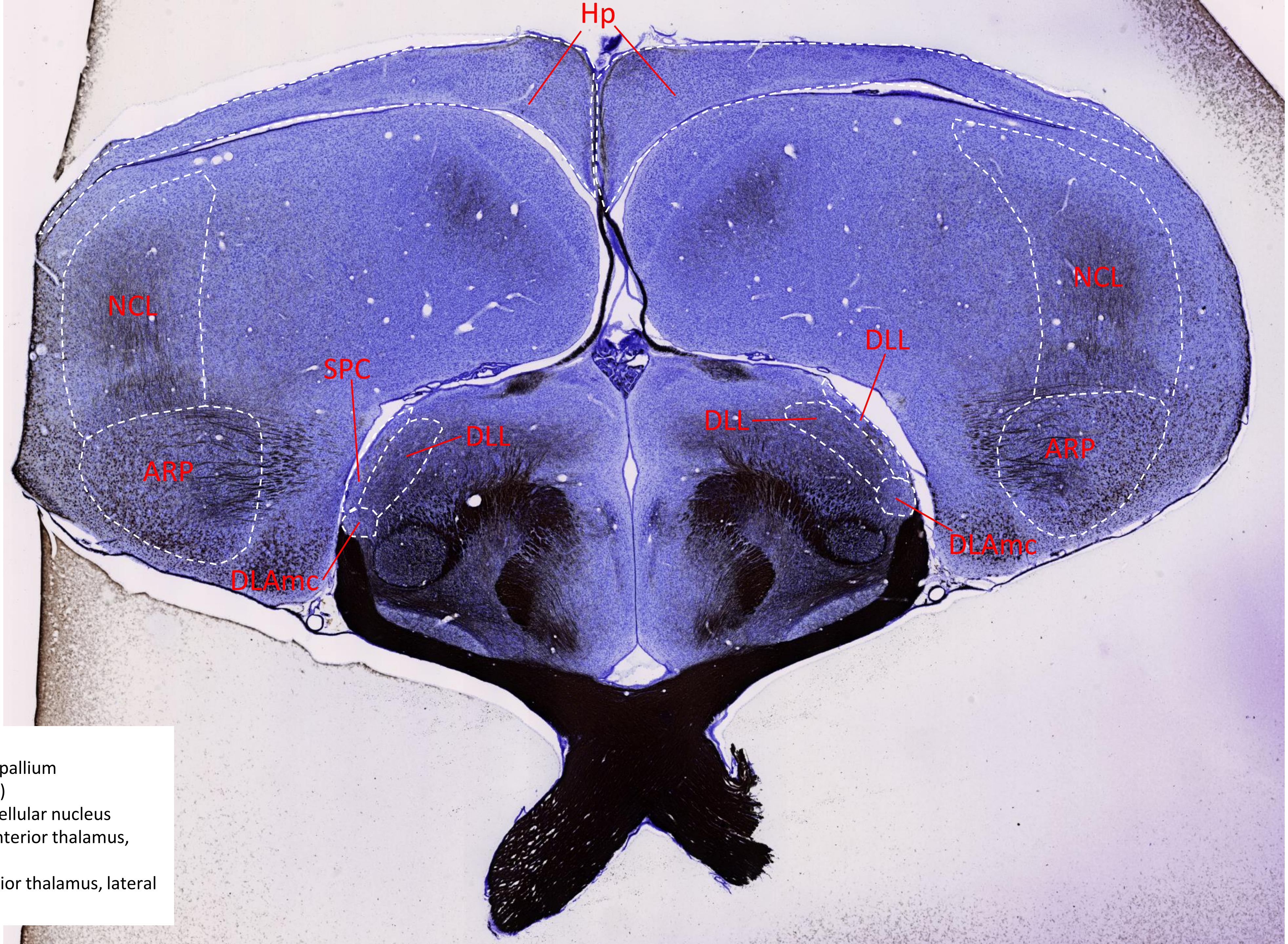

Hp: Hippocampal Area
NCL: caudolateral nidopallium
ARP: arcopallium (core)
SPC: superficial parvocellular nucleus
DLAmc: dorsolateral anterior thalamus, magnocellular part
DLL: dorsolateral anterior thalamus, lateral part

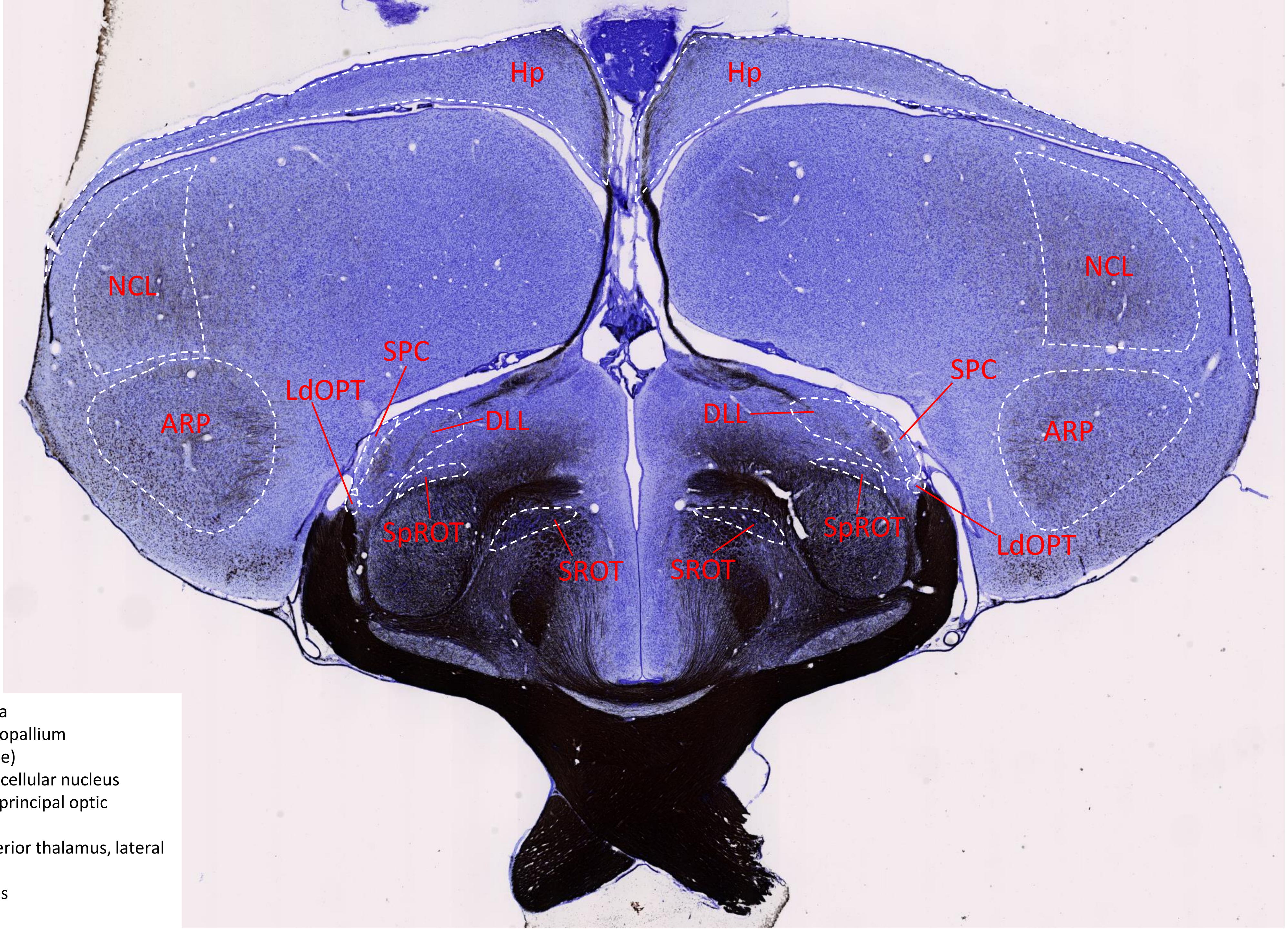

Hp: Hippocampal Area
NCL: caudolateral nidopallium
ARP: arcopallium (core)
SPC: superficial parvocellular nucleus
LdOPT: lateral dorsal principal optic thalamus
DLL: dorsolateral anterior thalamus, lateral part
SpROT: suprarotundus
SROT: subrotundus

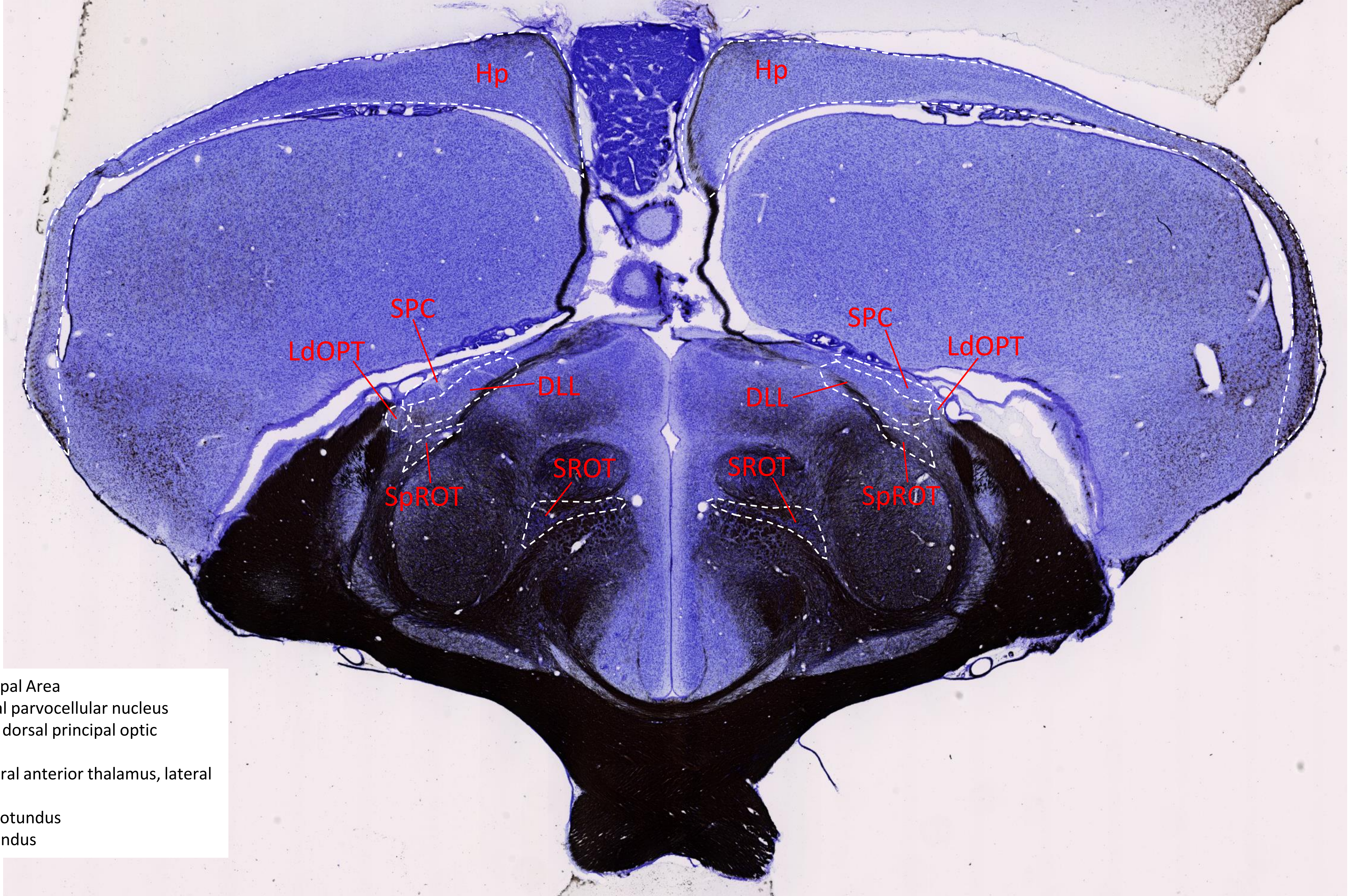

Hp: Hippocampal Area
SPC: superficial parvocellular nucleus
LdOPT: lateral dorsal principal optic thalamus
DLL: dorsolateral anterior thalamus, lateral part
SpROT: suprarotundus
SROT: subrotundus
