# Supplementary material for: A histological and diceCT-derived 3D reconstruction of the avian visual thalamofugal pathway
Source: Sci Rep. 2024 Apr 11;14:8447. doi: 10.1038/s41598-024-58788-z (PMC11006926; doi:10.1038/s41598-024-58788-z)
Supplement: Supplementary file 5 — Supplementary Information 5. [file 41598_2024_58788_MOESM5_ESM.docx]

| Abbreviation | Structure | Color |
| --- | --- | --- |
| DLAlr | dorsolateral anterior thalami, pars rostrolateralis |  |
| DLAmc | dorsolateral anterior thalami, pars magnocellularis |  |
| DLL | dorsolateral anterior thalami, pars lateralis |  |
| LdOPT | dorsolateral principal optic thalami |  |
| SPC | superficial parvocellularis |  |
| SpROT | suprarotundus |  |
| SROT | subrotundus |  |

Table S1. Summary of important thalamofugal structures within the thalamus including the abbreviation, full name, and color.
