# Supplementary material for: A histological and diceCT-derived 3D reconstruction of the avian visual thalamofugal pathway
Source: Sci Rep. 2024 Apr 11;14:8447. doi: 10.1038/s41598-024-58788-z (PMC11006926; doi:10.1038/s41598-024-58788-z)
Supplement: Supplementary file 6 — Supplementary Information 6. [file 41598_2024_58788_MOESM6_ESM.docx]

Table S2. Summary of important thalamofugal structures within the pallium including the abbreviation, full name, and color.

| Abbreviation | Structure | Color |
| --- | --- | --- |
| HA | apical hyperpallium |  |
| IHA | intercalated nucleus of the apical hyperpallium |  |
| HI | intermediate hyperpallium |  |
| HD | densocellular hyperpallium |  |
| NFL | frontolateral nidopallium |  |
| NCL | caudolateral nidopallium |  |
| ARP | arcopallium |  |
| Hp | hippocampus |  |
